# Upper Limb Work-Related Musculoskeletal Disorders in Operating Room Nurses: A Multicenter Cross-Sectional Study

**DOI:** 10.3390/ijerph16162844

**Published:** 2019-08-09

**Authors:** Marco Clari, Giacomo Garzaro, Matteo Di Maso, Francesca Donato, Alessandro Godono, Mario Paleologo, Valerio Dimonte, Enrico Pira

**Affiliations:** 1 Department of Public Health and Pediatrics, University of Turin, 10126 Turin, Italy; 2 Department of Clinical Sciences and Community Health, Branch of Medical Statistics, Biometry and Epidemiology “G.A. Maccacaro”, Università degli Studi di Milano, 20133 Milan, Italy; 3 Directorate of Allied Health Professionals, Città della Salute e della Scienza di Torino University Hospital, 10126 Turin, Italy

**Keywords:** scrub nurse, musculoskeletal disorders, ergonomic, operating room, occupational exposure, occupational medicine

## Abstract

This study aimed to evaluate the association between personal and job characteristics and the risk of upper limb work-related musculoskeletal disorders (WMSDs) among operating room nurses (ORNs). To this end, we collected data from 148 ORNs working at 8 Italian hospitals and measured any upper limb disabilities experienced in the previous year using the Italian version of the disabilities of the arm, shoulder and hand (DASH) questionnaire. The associations between personal and job characteristics and risk of upper limb WMSDs were estimated by unconditional logistic regression models. The prevalence of upper limb WMSDs was 45.9%. Multivariate analysis showed the “female gender” and “monthly hours spent working as a scrub nurse” to be directly associated with a higher DASH score (adjusted OR for gender = 5.37, 95% CI: 1.65–17.51, *p* < 0.01; adjusted OR for monthly hours as scrub nurse = 3.09, 95% CI: 1.33–7.19, *p* < 0.01). Overall, our findings indicate that a full-time job (>120 h/month) as a scrub nurse significantly increases the risk of developing upper limb WMSDs among female ORNs. Thus, to reduce such risk in this particularly sensitive population, we recommend urgent implementation of ergonomic interventions on surgical equipment alongside job rotation and medical surveillance programs.

## 1. Introduction

Work-related musculoskeletal disorders (WMSDs) are among the leading causes of occupational diseases among healthcare professionals worldwide [1,2]. WMSDs inevitably lead to increased rates of job turnover [3] and long sick leave [4], representing a social and economic burden due to long-term disability and decreased work efficiency [5]. WMSDs include all injuries involving the locomotor system, especially muscles, joints, ligaments, tendons and nerves. Even though lower back pain is the most commonly reported health issue, WMSDs quite frequently affect other body regions such as the upper limbs [6].

Nurses represent the largest professional group in the healthcare system with a higher prevalence of WMSDs compared to other health care professionals [7], ranging from 30% to 88%, depending on which body area is being considered [8]. Occupational factors contributing to WMSDs include physical and psychological workload [9], patient manual handling and job strain [10]. Moreover, long work hours, on-call, mandatory overtime and working on days off significantly increase musculoskeletal issues, mainly due to an increased exposure to physical demands [11]. In addition, socio-demographic factors and anthropometric measures are known to increase the risk of WMSDs in nursing personnel. In particular, women and overweight or obese individuals are at higher risk of WMSDs [12,13]. On the other hand, age is inversely associated with the risk of WMSDs [14].

In the operating room, nurses could work as scrub nurses that directly assist the surgeon, or circulating nurses that maintain a sterile environment, assist scrub nurses and take care of the patient before and after the operation. The prevalence of WMSDs among operating room nurses (ORNs) is generally higher compared to that of nonspecialized nurses [15]. This is particularly true for scrub nurses that being actively involved in creating and maintaining the surgical field and passing medical equipment to surgeons are more prone to WMSDs. In this regard, the peculiarities of their work tasks, such as continuous repetitive movements or unusual motions, the adoption of static and awkward postures for prolonged periods of time and lifting and holding up heavy surgical instruments when assisting the surgeon and caring for the patient, have been shown to play a crucial role in the development of WMSDs [16,17]. Accordingly, ORNs have generally an even higher risk of developing WMSDs if working full-time as scrub nurses [18].

Despite its widespread prevalence, the risk of upper limb WMSDs among ORNs has been scantily investigated, with a limited number of studies using inadequate measures [19] or failing to take into account the association between the activities performed by nurses in different settings and the risk of WMSDs [20]. To the best of our knowledge, there are no prior studies in the literature assessing the occupational and health implications of the time spent working as a scrub nurse with regard to upper limb WMSDs. 

Thus, the aim of this study was to evaluate the association between personal and job characteristics and the risk of upper limb WMSDs among ORNs, which represents an urgent unmet health need for these health care professionals

## 2. Materials and Methods 

### 2.1. Study Design and Data Collection

Between April and September 2018, we conducted a cross-sectional study on 148 ORNs working at 8 representative hospitals in Northwest Italy, with an overall response rate of 74%. The sample size required for an expected upper limb WDSDs proportion of 0.5, a finite population of 200 ORNs and a precision of 0.05 was 132 subjects. ORNs were approached in different hospital networks, such as hub, spoke and community hospitals. We considered nurses to be ORNs if they had been working in an operating room directly assisting surgeons (scrub nurses) or supporting anesthesiologists and all activities performed during surgery (circulating nurses). All nurses with at least five years of seniority were deemed eligible for the study. ORNS working part-time in other services, such as intensive care units, were excluded as well as nurses with daily or weekly hire. As nurses could shift between the scrub and circulation position, the cut-off value to define a full-time scrub nurse position was 120 working hours per month. This cut-off was chosen considering an average absence from work of 20 to 30 h/month due to holidays, sick leaves, training and other leaves of absence. 

### 2.2. Instruments

Information on socio-demographic factors (i.e., gender and age), job characteristics (i.e., number of years on the job as an ORN, number of monthly hours worked as a scrub nurse and type of hospital network and surgical specialty) and clinical data (i.e., previous episodes of upper limb pain, localizations of upper limb discomfort, familial predisposition to upper limb issues) were collected through a self-administered questionnaire. As WMSDs have an intermittent pattern of recurrence, the level of upper limb discomfort experienced by study participants in the previous year was measured as well [21]. To size the extent of upper limb WMSDs, the Italian version of the disabilities of the arm, shoulder and hand (DASH) questionnaire was used [22]. This self-report 30-item questionnaire measures the degree of problems in performing activities related to arm, shoulder and hand movements. It also evaluates activity-related symptoms and the impact of the disabilities on the psychosocial domain. Each item has a response on a scale from 1 (no difficulty or no symptom) to 5 (unable to perform activity or severe symptoms), providing a standardized upper extremity specific outcome ranging from 0 (no disability) to 100 (severe disability). The DASH was chosen as a single outcome measure for different upper limb disabilities as upper limbs could be considered a single functional unit [23]. Furthermore, to reduce the administrative burden of using many disease-specific outcome measures, a unique tool capable of comparing different upper limbs conditions in terms of health burden was considered preferable [24]. In addition, the DASH was selected between other patient-reported outcome measures such as the Neck and Upper Limb Index, the Upper extremity functional Index, and Upper Extremity Function Scale, as it was the only instrument capable of capturing the functional status of the upper limbs with a link with the International Classification of Functioning, Disability, and Health (ICF) [25]. In previous studies, the DASH showed good internal consistency (Cronbach’s alpha ≥ 0.90) and construct validity, proving to be an invaluable instrument to measure the health status of people suffering from upper limb disabilities and to facilitate study results comparisons worldwide [24]. In particular, in our sample the DASH reached a Cronbach’s α = 0.97. ORNs were considered to suffer from upper limb WMSDs if they reported a DASH score higher than 10.1, according to previous findings by Hunsaker et al. (2012) on an U.S. population of 1706 subjects [26]. Missing data were completely at random (Little’s test *p* > 0.05).

### 2.3. Statistical Analysis

Descriptive statistics analyses were reported as absolute frequencies and percentages for categorical variables and as means and standard deviations (SDs) for continuous variables. The strength of the linear relationship between variables was evaluated by Pearson correlation coefficient (*r*). ORNs were gathered in two groups according to the presence of upper limb WMSDs based on their DASH score. Odds ratios (ORs) and corresponding 95% confidence intervals (CIs) for the association between socio-demographic factors, job characteristics, other relevant variables and the risk of upper limb WMSDs were estimated by means of unconditional logistic regression models. ORNs were gathered in two groups according to the presence of the upper limb WMSDs based on their DASH score. The candidate model to evaluate the association between independent variables and a binary response variable (presence of upper limb WMSDs) was the logistic regression model. The multivariate model included terms for gender, age (<50, ≥50 years), years spent working as an ORN (<20, ≥20 years), monthly hours spent working as a scrub nurse (<120, ≥120 h) and surgical specialty (i.e., soft or hard). Our surgical specialty classification was based on the frequency of surgical type, surgical instruments used, surgery length and scrub nurse position [4,15,16,26]. In particular, soft surgical specialty included plastic, vascular, neurosurgery, otorhinolaryngology and ophthalmology, whereas hard surgical specialties included general surgery, orthopedic, urology and gynecology. All analyses were performed using the SAS software, version 9.4 (SAS Institute, Inc. Cary, NC, USA).

### 2.4. Ethical Approval

The study research protocol was first approved by the health directorate board of the Maggiore della Carità di Novara University Hospital (Protocol No.8179/18) and then by each of the health directorate board of the hospitals involved in the study. The research was conducted in accordance with the 1964 Declaration of Helsinki. Participation in the study was on a voluntary basis. After full disclosure about the purposes and procedures of the study, participants were requested to provide their written consent. Data were anonymized and treated confidentially. 

## 3. Results

Overall, ORNs were mainly women (79%) with an average age of 48.0 (±6.1) years (Table 1). Nurses had been working in an operation room for an average of 19.4 years (±8.7) and 108.1 h (±49.4) per month. Almost seventy percent of the ORNs (68.2%) worked at hub hospitals in general surgery (28.9%) and orthopedic (26.9%) specialties. Approximately one in two nurses (48.3%) had experienced one or more episodes of upper limb pain, especially arm/shoulder (36.1%) and hand (12.5%) over the previous year. The DASH score had a mean value of 14.0 (±17.0) and was positively but weakly correlated with the monthly number of hours spent working as a scrub nurse (*r* = 0.15), whereas it was negatively correlated with female gender (*r* = −0.20) (Table 2).

Almost half of the ORNs (45.9%) presented with upper limb WMSDs (DASH score > 10.1; Table 3). On univariate analysis, female nurses showed a three-fold increase in the risk of developing upper limb WMSDs (OR = 3.15, 95% CI: 1.26–7.87, *p* = 0.01). Likewise, working for more than 120 h per month as a scrub nurse doubled such risk (OR = 2.63, 95% CI: 1.25–5.52, *p* = 0.01). Age and surgical specialty increased the risk, albeit not significantly. Multivariate analysis showed the two variables “female gender” and “monthly hours spent working as a scrub nurse” to increase the risk of upper limb WMSDs (adjusted OR for female gender = 5.37, 95% CI: 1.65–17.51, *p* < 0.01; adjusted OR for >120 monthly hours as scrub nurse = 3.09, 95% CI: 1.33–7.19, *p* < 0.01; Table 3).

## 4. Discussion

In an ORN population, we show that the two variables “female gender” and “monthly hours spent working as a scrub nurse” are directly associated with increased risk of developing WMSDs. This observation is particularly relevant if we consider that among ORNs women represent the majority of workers.

Our findings are in good agreement with previous evidence indicating that female nurses, especially scrub nurses, working in the operating room have a higher prevalence of upper limb disabilities [18,27]. Specifically, we show that full-time scrub nurses are three times more likely to develop upper limb issues than ORNs rotating between different job tasks. In contrast with other studies [28], ORN seniority does not appear to be associated with WMSD risk. Likewise, no association was found with regard to type of surgical specialty or hospital. Possible explanatory factors associated with the increased risk of WMSDs in women might be the gender specific somatic, hormonal and psychological differences. Women are more prone to WMSDs in cold working environments [29] and differences are also present in the methods, more precisely, the sequential routinely procedures used to perform the same task [30]. Furthermore, women are generally more in charge of the domestic work especially in familiar contexts, increasing the musculoskeletal demands with reduced recovery time [31] living also less opportunity for strengthening body muscles and having higher risks of overweight consequences [32]. Thus, the planning of gender-tailored specific prevention and protection measures could be of help in reducing WMSDs burden in ORNs. In our sample a healthy worker effect bias could have influenced the results. Nurses with higher WMSDs could have exit from their ORN position leaving healthier nurses in the workforce. The result of this effect could have led to a downward bias in the association between cumulative exposure and disease [33]. Moreover, ORNs with a higher expertise could have learnt during time how to adapt their work tasks to be ergonomically efficient. The type of surgical specialty and hospital could have had a lower influence on upper limb WMSDs than contextual factors, such as surgical operation type and single operating room organization.

Our findings are particularly relevant when we consider that scrub nurses are continuously exposed to both physical and temporal risk factors, such as low temperature, high force, highly repetitive tasks and use of vibrating instruments, all potential determinants of work-related upper extremity cumulative disorders. Furthermore, scrub nurses must stay in the operating room at all times during the surgical procedure, which causes prolonged static and awkward postures, constant trunk flexion and upper extremity strain [34]. In this regard, a prolonged static position is generally recognized to be more harmful than a dynamic posture due to a higher levels of lactic acidosis and toxins caused by the former [35].

Indeed, scrub nurses constantly move their gaze from the patient to the surgeon so as to be ready to hand immediately the right surgical tool at the surgeon’s request. Thus, nurses are subject to repetitive manipulative tasks, such as reaching for supplies and passing instruments, that put excessive strain on their upper limbs [36]. In doing so, scrub nurses bend their wrists and hold their elbows higher than they should, thereby overloading muscles and joints. Therefore, it is advisable that scrub nurses should maintain the joint excursion in a neutral zone where the height of their elbows lies between a factor of 0.7–0.8, below their shoulders [37]. Furthermore, an upper extremity support should always be used in order to minimize physical pressure, discomfort and bruises deriving from inappropriate positioned surfaces, such as instrument trays or operating tables [38].

In this scenario, the poor ergonomic design of surgical instruments also appears to contribute to the increased risk of WMSDs [16]. Surgical instruments are indeed seldom designed according to sound ergonomic principles that would ease their handling by health care providers. The ergonomic conditions of the operating room and surgical field, in addition to the ergonomic design of surgical instruments can be an important factor in the appearance of upper limb WMSDs. Surgical instruments makers should consider involving professional workers into the design of surgical instruments to include also users experience. Moreover, healthcare professionals working in the operating room, independently of their clinical role, should be engaged in the design and redesign of operating room to create a user-centered work environment. Furthermore, poorly designed instruments can in fact increase finger, hand and wrist strains of operators, making these subjects more likely to develop upper limb disabilities (e.g., arthritis, sprains, dislocations and fractures). Thus, a more operator-friendly design would definitely help reduce the physical strain for scrub nurses and surgeons in the operating room. This would be even more important for people with smaller hands, like women, dealing with heavy and large instruments that, according to a recent study, are more at risk of developing upper extremities paresthesia [39].

In light of the above, it is quite clear that a careful evaluation of the physical strain endured by scrub nurses would favor the implementation of occupational health measures aimed at reducing the risk of WMSDS. It is therefore essential to devise innovative technical measurements capable of evaluating objectively the physical workload to then determine specific ergonomic risk factors. Since motion sensors and electrodes could interfere with operators’ activities, a task analysis seems at present a more viable approach to carry out this type of assessment. Moreover, a task analysis would allow evaluating type-specific surgical processes based on a holistic, worker-centered approach taking into account all most relevant components of human skills [40].

Based on our findings, we propose several strategies to overcome WMSDs in ORNs. First and foremost, the simple introduction of a job rotation strategy could be used as an administrative action. In this regard, a recent study has shown that the introduction of job rotations can contribute to increasing muscular activity variability, reducing the burden of occupational injuries among scrub nurses [41]. Despite the evidence that work organization factors can influence the likelihood of developing WMSDs [42], international regulatory authorities have yet to issue any recommendation in this regard. To reduce upper limb WMSDs in this specific population, it would probably be enough to just promote a part-time position as a scrub nurse rotating between different tasks in the operating room, even though this job rotation would likely be less feasible for some types of surgery where a high specialization level is required. Nonetheless, this strategy could be viewed as an opportunity for experienced nurses to hand down their skills to their junior colleagues, at a time when the nurse population is aging and the senior staff are gradually becoming less involved in scrub nurse activities [43]. Another fairly simple and feasible solution to increase work-rest ratio and mitigate task-related fatigue in ORNs would be that of incorporating intraoperative microbreaks with exercise that could be routinely performed in the sterile field of the operating room [44].

In general, specific ergonomic training should be implemented for ORNs and scrub nurses apart from patient manual handling. This training should teach how to adopt a correct posture in the operating room and how to properly place surgical instruments to create an appropriate operating room working environment. Moreover, as the operating room has also high psychological demands [45], prevention interventions should also consider the psychological factors of the work environment. Therefore, surgical subspecialties should develop context-specific interventions tailored to daily practice.

Lastly, as physical activity may represent a resource to moderate musculoskeletal morbidity in the working population [46], improve the physical fitness of ORNs could be recommended. Thus, multicomponent interventions combining ergonomic with healthy lifestyles such as physical fitness and behavioral interventions should be considered in this special population [47].

One limitation of our study is the use of a self-reported outcome measure that, even if apparently sound, could be subject to recall bias. However, using the validated Italian version of the DASH questionnaire should have minimized this bias. The cross-sectional design is another limitation of the present study. Although the cross-sectional design is suitable for estimating upper limb WMSDs prevalence among ORNs, it fails to estimate the causal relationship between factors considered and the risk of WMSDs. However, it is likely that WMSDs could have modified the behaviors of ORNs while it is unlikely that WMSDs could have increased the “monthly hours spent working as a scrub nurse”.

Another limitation of our study is that, albeit the response rate was good, a non-response analysis could not be performed due to lack of non-respondent data. Consequently, a bias due to selective non-response cannot be ruled out. For these reasons, our results should be interpreted with caution.

## 5. Conclusions

Altogether, our findings indicate that ORNs exposed to a scrub nurse full-time job are three times more likely to present with upper limb WMSDs compared to ORNs working less than 120 h/month as scrub nurses. We also show that the risk of WMSDs in the ORN population is higher among female nurses. Given that women represent the vast majority of ORNs, our findings call for the urgent implementation of ergonomic interventions aimed at reducing the risk of developing WMSD in this sensitive population.

Additional longitudinal studies using instrumental procedures to diagnose upper limb WMSDs after an initial screening with a self-reported outcome measure questionnaire are warranted to further corroborate the determinants of WMSDs among female scrub nurses. Furthermore, it will be crucial to assess how psychological demands contribute to the development of WMSDs, and how these physical disabilities influence the ORN work-related quality of life.

## Figures and Tables

**Table 1:** Distribution of 148 operating room nurses (ORNs) according to socio-demographics factors, job characteristics, body district pain and disabilities of the arm, shoulder and hand (DASH) score. Italy, 2018.

Variables	
**Gender (*n*; %)**	
Men	31 (21.0)
Women	117 (79.0)
**Age: years (mean; SD)**	48.0 (6.1)
**Years as ORN (mean; SD)**	19.4 (8.7)
**Monthly hours as scrub nurse ^†^ (mean; SD)**	108.1 (49.4)
**Hospital network (*n*; %)**	
Hub	101 (68.2)
Spoke	13 (8.9)
Community	34 (23.0)
**Surgical specialty ^†^ (*n*; %)**	
Soft	34 (23.5)
Plastic	10 (6.9)
Vascular	9 (6.2)
Neurosurgery	8 (5.5)
Ophthalmology	4 (2.8)
Otorhinolaryngology	3 (2.1)
Hard	111 (76.5)
General surgery	42 (28.9)
Orthopedic	39 (26.9)
Gynecology	16 (11.0)
Urology	14 (9.7)
**Episodes of previous upper limb pain ^†^ (*n*; %)**	
No	76 (51.7)
Yes	71 (48.3)
Arm/shoulder	52 (36.1)
Multiple	23 (16.0)
Hand	18 (12.5)
Neck	8 (5.6)
Elbow	7 (4.9)
Wrist	6 (4.2)

Note: ^†^ Presence of missing values. SD = Standard deviation.

**Table 2:** Correlation between selected socio-demographic factors, job characteristics and disabilities of the arm, shoulder and hand (DASH) score of 148 operating room nurses (ORNs). Italy, 2018.

Variables	DASH Score
Gender	−0.20
Age	0.07
Years as scrub nurse	−0.03
Years as ORN	−0.01
Monthly hours as scrub nurse	0.15
Hospital network	−0.04

**Table 3:** Odds ratios (ORs) and 95% confidence intervals (CIs) for disabilities of the arm, shoulder and hand (DASH) score according to socio-demographic factors and job characteristics among 148 operating room nurses. Italy, 2018.

Variables	Upper Limb Work-Related Musculoskeletal Disorders				Univariate	*p*-Value	Multivariate ^‡^	*p*-Value
	Yes (*n* = 63)		No (*n* = 85)					
	*n*	(%)	*n*	(%)	OR (95% CI)		OR (95% CI)	
Gender								
Men	7	(11.1)	24	(28.2)	1 ^♦^		1 ^♦^	
Women	56	(88.9)	61	(71.8)	**3.15** (1.26–7.87)	*p* = 0.01	**5.37** (1.65–17.51)	*p* < 0.01
Age (years)								
<50	37	(58.7)	57	(67.1)	1 ^♦^		1 ^♦^	
≥50	26	(41.3)	28	(32.9)	1.43 (0.73–2.81)	*p* = 0.30	1.04 (0.42–2.57)	*p* = 0.93
Years as operating room nurse								
<20	34	(54.0)	45	(52.9)	1 ^♦^		1 ^♦^	
≥20	29	(46.0)	40	(47.1)	0.96 (0.50–1.84)	*p* = 0.90	1.10 (0.46–2.60)	*p* = 0.83
Monthly hours as scrub nurse								
<120	16	(25.4)	42	(49.4)	1 ^♦^		1 ^♦^	
≥120	35	(55.6)	35	(41.2)	**2.63** (1.25–5.52)	*p* = 0.01	**3.09** (1.33–7.19)	*p* < 0.01
Surgical specialty ^§^								
Soft	11	(17.5)	23	(27.1)	1 ^♦^		1 ^♦^	
Hard	50	(79.4)	61	(71.8)	1.71 (0.76–3.85)	*p* = 0.19	1.05 (0.40–2.73)	*p* = 0.92

Note: Cut-off for DASH score was 10.1; ^‡^ Mutually adjusted; ^♦^ Reference category; ^§^ Soft surgical specialties includes vascular, otorhinolaryngology, ophthalmology, plastic, and neurosurgery; hard surgical specialties include general surgery, urology, orthopedic, and gynecology; in bold significant results.
